# A Cross-Sectional Survey to Identify Sociodemographic Factors Associated with the Frequency of Urinalysis in a Representative Sample of Adults in Poland, 2024

**DOI:** 10.3390/healthcare12151475

**Published:** 2024-07-25

**Authors:** Gabriela Moczeniat, Mateusz Jankowski, Aneta Duda-Zalewska, Mariusz Gujski

**Affiliations:** 1 Department of Public Health, Medical University of Warsaw, 02-097 Warsaw, Poland; 2 Department of Urology, Mazovia Hospital Warsaw, 02-797 Warsaw, Poland; 3 School of Public Health, Centre of Postgraduate Medical Education, 01-826 Warsaw, Poland

**Keywords:** urinalysis, urologic diseases, laboratory testing, diagnostics, medical procedures utilization, urology, Poland

## Abstract

A general urine test is considered one of the basic diagnostic tests using in healthcare. This study aimed to analyze sociodemographic factors associated with the frequency of urine testing in Poland. This cross-sectional survey was conducted using computer-assisted web interviewing (CAWI) between 1 March and 4 March 2024. A representative sample of 1113 adults in Poland (aged 18–86 years, 52.5% of whom were females) took part in the study. The survey showed that 46.3% of adults in Poland had a urinalysis in the last 12 months. One-fifth (20.7%) of the participants had a urinalysis more than a year ago but not more than 2 years ago. Moreover, 26.7% had a urinalysis performed 2–3 years ago. Among all participants, female gender (OR = 1.31 [1.01–1.68]; *p* < 0.05), being aged 70 years and over (OR = 2.22 [1.23–4.02]; *p* < 0.01), having children (OR = 1.45 [1.01–2.09]; *p* < 0.05), and having urologic diseases (OR = 2.34 [1.79–3.02]; *p* < 0.001) were significantly associated with having urinalysis in the last 12 months. Among respondents without urologic diseases, female gender (OR = 1.33 [1.02–1.74]; *p* < 0.05), being aged 60 years and over (*p* < 0.05), and being married (OR = 1.45 [1.09–1.94]; *p* < 0.05) were significantly associated with having a urinalysis in the last 12 months. There was no significant impact of educational level, occupational status, or financial situation on the frequency of urinalysis.

## 1. Introduction

Urinalysis (also called a urine test) is one of the most common diagnostic tests used in medical practice [1]. This test includes physical, chemical, and microscopic examinations of a sample of urine [1,2,3]. A variety of compounds can be analyzed in urine samples [1,2,3,4]. Samples are usually collected in the morning from the midstream in a completely clean (sterile) container [3,4]. The collected sample should be transported to a laboratory or dedicated medical point without undue delay [5].

Urinalysis is a simple diagnostic test used for screening and monitoring of therapy and progress of urologic diseases [2,4]. Urinalysis is commonly used to detect and monitor urologic diseases [3,6]. However, it can also detect advanced stages of diabetes and liver diseases [7,8]. General practitioners widely use urinalysis mostly to screen or monitor kidney diseases, urinary tract infections (UTIs), diabetes, and liver diseases [6,7,8].

Physical examination of urine samples includes color and appearance assessment [2,3]. Medications and diet influence urine color, so patients’ education on dietary-related behaviors (e.g., avoiding certain types of foods that may change the color of urine) is an important part of urine testing [9]. Dehydration, UTIs, kidney stones, urinary tract disorders, and diabetes are the most common causes of abnormalities in urine color and appearance [3,7,8,9].

Chemical examination of urine samples includes testing for a variety of chemical compounds that can be found in urine samples, including the presence of proteins (mostly albumins), which indicates kidney failure, heart failure, or dehydration; pH level, which may be related to UTIs or diabetes; ketones and glucose, which are related to diabetes and its complications; bilirubin, related to liver diseases; and leukocytes, which are mostly observed in UTIs or urinary tract inflammation [3,10].

Microscopic examination of urine samples is mostly focused on the detection of red blood cells, white blood cells, epithelial cells, urinary casts, and the presence of bacteria or parasites [3,11]. Microscopic examination allows detection of urinary tract issues that may cause bleeding or UTIs [12].

Nationwide data on the frequency of urinalysis may reflect public attitudes to preventive screening as well as the diagnostic practices of healthcare workers [13,14]. In recent years, urinalysis has been considered a preventive test in healthy adults [3]. According to the European Urinalysis Guidelines published by the European Federation of Clinical Chemistry and Laboratory Medicine (EFLM) in 2023, urinalysis is not yet recommended as a screening test [15]. Despite these recommendations, urinalysis is often considered by physicians as a commonly used screening and diagnostic test. In Poland, urinalysis tests can be paid for by the patient, or the patient can be referred for urinalysis by a doctor. Moreover, urinalysis is often a part of screening diagnostic tests offered by private laboratories or is a part of individual medical insurance packages [2,4]. Urinalysis may also be performed as a part of screening tests ordered by occupational medical services [16] as well as national preventive screening programs addressed to adults aged 40 years and over [17]. In 2021, a nationwide “Prevention 40 plus” program was initiated in Poland [18]. This program provided free-of-charge access to basic blood-based screening tests, as well as urinalysis, and is one of the biggest health policy programs focused on early detection of diseases launched in Poland in the past decade.

Patients are also referred for urinalysis by a variety of healthcare professionals, including general practitioners, urologists, nephrologists, diabetologists, and internal medical specialists to diagnose and monitor the progress of infections of the urinary tract, kidney diseases, diabetes, and liver diseases [1,2,3,4,7,8]. Data on the frequency of urinalysis may also inform clinicians and public health professionals about the current utilization of urinalysis and its actual role in the management of diseases.

Urinalysis is a simple test, but the patient is obligated to buy a container (usually in the pharmacy), collect the sample, store it, and transport it to the laboratory [5]. This process may influence the patient’s readiness to have a urinalysis. Previous studies showed that sociodemographic variables have an impact on public attitudes toward screening tests, including eye tests and blood-based tests [19,20]. Moreover, health inequalities in access to healthcare services (including laboratory diagnostics) may be observed in the case of urinalysis, as this test includes a series of procedures from sample collection to delivery to the laboratory point.

It is estimated that over 4 million people in Poland have kidney diseases [21]. The burden of urologic diseases in Poland is increasing, mostly due to population aging and lifestyle-related behaviors [22,23]. Many asymptomatic urologic and nephrological conditions may be diagnosed using a simple urinalysis. Urine tests play a role in secondary screening as a part of both self-paid testing and preventive actions undertaken by doctors. Moreover, urine tests are used as diagnostic tools by different specialists.

However, there is a lack of data on public attitudes toward basic urine testing in Poland, especially nationwide data on the frequency of urinalysis.

This study aimed to assess the frequency of urinalysis and to identify factors associated with the frequency of urinalysis among adults in Poland.

## 2. Materials and Methods

### 2.1. Study Design and Sample

A representative sample of 1113 adults in Poland took part in this cross-sectional survey. Data were collected using a computer-assisted web interview (CAWI) method between 1 and 4 March 2024. The study questionnaire included questions on urologic diseases, their prevention methods, and sociodemographic data and was available online on a dedicated web platform managed by a public opinion research agency [24] contracted for data collection. All study procedures were prepared by the research team. Respondents were selected from over 100,000 verified individual users available in the database of the public opinion research agency, and the stratification model included gender, age, and place of residence. Quota sampling was used. If selected individuals refused to complete the questionnaire, another participant was selected according to demographic characteristics. The questionnaire could only be completed once. The questionnaire could only be closed and saved when all questions had been answered.

The study population’s demographic structure was representative of Poland’s adult population, in line with the demographic data published by Statistics Poland [25]. All participants declared informed consent to take part in this survey, and the study protocol was approved by the Ethical Board of the Medical University of Warsaw (approval number: AKBE/43/2024).

### 2.2. Measures

The study’s questionnaire was developed by public health specialists, urologists, and healthcare management experts (Supplementary File S1).

A pilot study was carried out, and 8 adults (aged 19–63 years) completed the questionnaire twice, 7 days apart. After the pilot study, one question and three answers to multiple-choice questions were revised to improve the clarity of the questions.

Participants were asked to indicate the time since their last urine test with the question “When was the last time you had a urine test (passed urine for analysis)?”, to which there were six possible answers: in the last month; more than a month ago but not more than 12 months ago; more than a year ago but not more than 2 years ago; over 2 years ago but not more than 3 years ago; over 3 years ago; never.

Based on the following question: “In the last 6 months, have you had any urologic diseases (e.g., kidneys, bladder, prostate diseases, urinary tract infections) for which you consulted a doctor? (yes/no)”, the participants were divided into two groups: those with urologic diseases in the last 6 months and those without urologic diseases.

Respondents were asked: “How do you assess the financial status of our household? (good/moderate/bad)”. Currently employed or self-employed individuals were considered as those with active occupational status, and pensioners, students, and unemployed individuals were considered as those with passive occupational status.

### 2.3. Data Analysis

Data were analyzed with IBM SPSS version 29 (Armonk, NY, USA). Categorical variables were presented with frequencies and proportions. Cross-tabulations with chi-squared tests were performed to compare categorical variables. Logistic regression analyses were carried out to identify sociodemographic factors associated with the frequency of urinalysis (two separate analyses: urinalysis in the last 12 months and urinalysis in the last 3 years). Urinalysis in the last 12 months and urinalysis in the last 3 years were considered as dependent variables in the models. In multivariable logistic regression models, only variables that were statistically significant in univariable analyses were included. Multivariable logistic regression analyses were prepared separately (1) for all participants and (2) for those participants without urologic diseases in the last 6 months. The strength of associations was presented with odds ratio (ORs) and 95% confidence intervals (95%CIs). The statistical significance level was set at *p* < 0.05.

## 3. Results

### 3.1. Frequency of Urinalysis by Sociodemographic Factors

The study population included 1113 adults, aged 18–86 years, 52.5% of whom were females (Table 1). Among the participants, 14.6% had visited a doctor due to urologic diseases in the last 6 months.

Over one-tenth of participants had a urinalysis in the last 30 days, and 34.5% of participants had a urinalysis within the last 1–12 months (Table 1). One-fifth of participants had a urinalysis more than a year ago but not more than 2 years ago, and one-tenth had a urinalysis within the last 2–3 years (Table 1). Among the participants, 6.4% declared that they had never had a urinalysis. Among participants without urologic diseases in the last 6 months (*n* = 951), 9% had a urinalysis in the last month, 32.6% had a urinalysis within the last 1–12 months, 22% had a urinalysis more than a year ago but not more than 2 years ago, and 11.2% had a urinalysis within the last 2–3 years. Moreover, 18.3% of participants had a urinalysis over 3 years ago, and 6.8% declared that they had never had a urinalysis.

There were differences in the percentages of participants who self-reported urologic diseases that required a visit to a doctor in the last 6 months among age groups, with the highest percentage among the youngest adults aged 18–29 years (25.2%) and the lowest among those aged 50–59 years (11.4%; *p* = 0.01). There were no differences in the percentages of participants who self-reported urologic diseases for other sociodemographic variables.

There were statistically significant differences in the frequency of urinalysis for the sociodemographic factors, which are presented in Table 1.

### 3.2. Factors Associated with the Frequency of Urinalysis

Among all adults in Poland, female gender, being aged 70 years and over, having children, and having urologic diseases were significantly associated (*p* < 0.05) with having urinalysis in the last 12 months (Table 2). Moreover, female gender, being aged 60 years and over, having children, active occupational status (being employed or self-employed), and having urologic diseases were significantly associated (*p* < 0.05) with having urinalysis in the last 3 years (Table 2).

Among participants without urologic diseases, female gender, being aged 60 years and over, and being married were significantly associated (*p* < 0.05) with having urinalysis in the last 12 months (Table 3). Moreover, female gender, being aged 60 years and over, and living in cities with 100,000 to 499,999 residents were significantly associated (*p* < 0.05) with having urinalysis in the last 3 years (Table 3).

## 4. Discussion

This study provides nationwide data on the frequency of urinalysis. The findings from this study showed that less than half of adults in Poland had a urinalysis within the last year. Moreover, sociodemographic differences in the frequency of urine testing were observed, with female gender and older age (60 and over) being the most important factors associated with a higher frequency of urinalysis. There was no significant impact of educational level, occupational status, or financial situation on the frequency of urinalysis.

Kidney diseases, some metabolic diseases, and urinary tract disorders may be asymptomatic for a long time, so urinalysis is an important test that may detect several health issues at an early stage [2,3,6,7,8]. In Poland, urinalysis used to be a basic screening test along with blood count, glucose level testing, and lipid panel. Despite the European Urinalysis Guidelines [15], urinalysis is still available as a screening test in preventive programs in Poland. In this study, 46.3% of adults in Poland had a urinalysis in the last 12 months, which showed that urinalysis is commonly used in healthcare in Poland.

Urinalysis is widely available in Poland. Each general practitioner can refer patients for free-of-charge urinalysis [26]. Moreover, since 1 July 2021, there has been a nationwide preventive program called “Prevention 40 plus” aimed at the early detection of diseases among adults aged 40 years and over [18]. This program offers a set of laboratory tests, including urinalysis, that are free of charge and easily accessible—each adult aged 40 years and over can fill out an online questionnaire and obtain a referral for laboratory tests [18]. The testing can be performed in both public and private laboratories that are involved in the “Prevention 40 plus” screening program. Moreover, the price of urinalysis is estimated at EUR 3–5, so this test is also accessible as a paid out-of-pocket service.

The COVID-19 pandemic had an impact on the health behaviors of society and limited access to healthcare services, including preventive services [22,23,27]. Data on public attitudes towards preventive screening one year after the onset of the COVID-19 pandemic in Poland (October–December 2021) showed that 53.1% of Poles had a urinalysis in the last 12 months and that 20.2% had not had a urinalysis in the last 3 years [27]. This study was carried out four years after the confirmation of the first COVID-19 case in Poland in a representative sample of adults in Poland. The percentage of adults in Poland who had a urinalysis in the last 12 months was lower than the datum reported in 2021 (46.3% vs. 53.1% [27]). Moreover, in this study, 22.5% had not had a urinalysis in the last 3 years, which is a higher percentage than that reported in the study from 2021 (20.2%) [27]. This observation suggests that the COVID-19 pandemic increased the level of public interest in preventive services and health-related issues, and this interest decreased with the decrease in health emergencies resulting from COVID-19 [28].

In this study, analysis of sociodemographic factors associated with the frequency of urinalysis was conducted in two ways: among all adults in Poland as well as among those without urologic diseases that required a visit to a doctor in the last 6 months. This approach was selected to exclude individuals who had a urinalysis due to urologic conditions and provide more precise data on preventive urinalysis.

Female gender and older age (60 years and over) were the most important factors associated with the highest frequency of urinalysis among all adults in Poland as well as among those without urologic diseases. Older age is a significant risk factor for urologic diseases [29,30]. Elderly individuals’ having the highest frequency of urinalysis may be a result of the higher risk of urologic diseases in this group. Females are at higher risk of UTIs [31], but males are at higher risk of serious urologic diseases, including cancers (e.g., bladder cancer) [32]. In this study, female gender was associated with a higher frequency of urinalysis, both among all adults as well as those without urologic diseases. This observation may be a result of the fact that females are at higher risk of urinary tract infections (UTIs) and that urinalysis is performed as a diagnostic test [33]. Moreover, urinalysis is often performed in pregnant women as a screening test. Nevertheless, males should be educated by physicians on the role of urinalysis in the screening and diagnosis of urological conditions.

Among all adults in Poland, visiting a doctor due to urologic diseases in the last 6 months was associated with a higher frequency of urinalysis. This observation confirms that urinalysis is still an important part of diagnostic tests for urologic diseases [1,2,3,4]. However, further studies are needed to assess the role of urinalysis in medical diagnostics and the behavior of healthcare professionals related to the types of diagnostic tests recommended to patients. In this study, having children was also associated with the highest frequency of urinalysis both in the last 12 months and the last 3 years. It can be hypothesized that having children may be associated with behaviors related to health prevention and screening willingness among adults.

Among those without urologic disease, only three sociodemographic factors were associated with the frequency of urinalysis. Except for female gender and older age, being married was associated with the highest frequency of urinalysis in the last 12 months. This observation may be related to group behaviors, like participation in preventive programs (e.g., “Prevention 40 plus”) by both spouses [18]. Moreover, living in cities with 100,000 to 499,999 residents was associated with a higher frequency of urinalysis in the last 3 years. This observation may be a result of the distribution of medical laboratories or healthcare facilities within medium-sized cities and shorter distances from places of residence to medical laboratories. This observation suggests that access to medical laboratories and diagnostics in rural areas and small cities still poses a challenge for health policymakers in Poland.

In multivariable logistic regression models, educational level (having been through higher education) is often associated with the highest level of health awareness and higher health literacy levels [34]. However, in this study, there was no impact of educational level on public attitudes towards the frequency of urinalysis. Moreover, economic status [35] was not significantly associated with the frequency of urinalysis, which may be a result of the wide access to free-of-charge urinalysis and its lower price as a commercial service. There was no impact of place of residence [36] on compliance with guidelines on annual urinalysis, which may be a result of the high accessibility of this screening test. There was also no impact of occupational status [37] on the frequency of urinalysis, which may be due to the fact that urinalysis is not considered a basic laboratory test during initial or follow-up check-ups within occupational medical services.

The data presented in this study provide information on public attitudes to preventive screening for urologic diseases in Poland as well as the utilization of urinalysis by healthcare professionals in Poland. The findings presented in this study may help healthcare providers in Poland to notice which groups of people are currently less likely to undergo urinalysis and to educate and involve them in the national screening program. This study showed sociodemographic differences in the frequency of urinalysis, especially by gender and age. Males are at higher risk of urologic diseases [32], but this study showed a lower frequency of urinalysis among males compared to females. An educational campaign targeted at the general population should underline the role of urinalysis in urologic disease prevention and early detection. Moreover, healthcare professionals should be educated on current guidelines on the use of urinalysis as a diagnostic test and updated guidelines on the delisting of urinalysis from common screening tests [15]. National guidelines and health policy programs (including the “Prevention 40 plus” program) should be revised to comply with the European recommendations [15].

This study was limited to urinalysis, and other diagnostic or screening tests used in urology were not included. However, urinalysis is the cheapest and most accessible screening test for urologic diseases. Further studies should also include more advanced diagnostic tests, like ultrasound of the urinary tract. Second, in this study, the overall frequency of urinalysis in a representative sample of adults in Poland was assessed, without consideration of different screening tests (self-paid or those included in the “Prevention 40 plus” program) or diagnostic tests ordered by physicians as a part of diagnostic procedures for kidney or urological diseases. Due to the study design and the questions used in this study (especially the question about visiting a doctor due to urologic diseases in the last 6 months), there was no clear way of differentiating those individuals who underwent urinalysis as part of a screening and those participants who may have undergone this test as part of a medical evaluation due to urological conditions that occurred more than 6 months ago. The findings from this study may, however, provide information about the general frequency of urinalysis among the adult population of Poland for both screening purposes and medical conditions that has implications for the healthcare system in Poland. This was a cross-sectional survey, so recall bias may have occurred. The data were self-reported, and electronic health records were not verified to assess the urinalysis tests of individual participants.

## 5. Conclusions

This study provided data on the frequency of urinalysis in Poland and its utilization in the healthcare system in Poland. Less than half of adults in Poland had a urinalysis in the last 12 months. Gender and age were the most important sociodemographic factors associated with the frequency of urinalysis among adults without urologic diseases. Urologic diseases (especially kidney diseases) are often asymptomatic, so there is a need to increase public awareness of urinalysis and its role in the early detection of urologic conditions. Moreover, physicians should be educated on the current screening recommendations and screening laboratory tests.

## Figures and Tables

**Table 1:** Frequency of urinalysis by sociodemographic factors among adults in Poland, March 2023 (*n* = 1113).

Variables	Study Sample	Time Since Last Urine Test among Adults in Poland According to Sociodemographic Factors						
		In the Last 30 Days	More Than a Month Ago but Not More Than 12 Months Ago	More Than a Year Ago but Not More Than 2 Years Ago	Over 2 Years Ago but Not More Than 3 Years Ago	Over 3 Years Ago	Never	*p*
	*n* (%)	*n* (%)	*n* (%)	*n* (%)	*n* (%)	*n* (%)	*n* (%)	
Overall		131 (11.8)	384 (34.5)	230 (20.7)	117 (10.5)	180 (16.2)	71 (6.4)	
Gender								
Female	584 (52.5)	72 (12.3)	215 (36.8)	125 (21.4)	70 (12.0)	85 (14.6)	17 (2.9)	<0.001
Male	529 (47.5)	59 (11.2)	169 (31.9)	105 (19.8)	47 (8.9)	95 (18.0)	54 (10.2)	
Age [years]								
18–29	173 (15.5)	17 (9.8)	44 (25.4)	27 (15.6)	21 (12.1)	30 (17.3)	34 (19.7)	<0.001
30–39	215 (19.3)	18 (8.4)	51 (23.7)	54 (25.1)	23 (10.7)	47 (21.9)	22 (10.2)	
40–49	185 (16.6)	19 (10.3)	55 (29.7)	44 (23.8)	25 (13.5)	34 (18.4)	8 (4.3)	
50–59	139 (12.5)	17 (12.2)	55 (39.6)	26 (18.7)	12 (8.6)	26 (18.7)	3 (2.2)	
60–69	266 (23.9)	37 (13.9)	111 (41.7)	54 (20.3)	25 (9.4)	37 (13.9)	2 (0.8)	
70 and over	135 (12.1)	23 (17.0)	68 (50.4)	25 (18.5)	11 (8.1)	6 (4.4)	2 (1.5)	
Education								
Primary	22 (2.0)	3 (13.6)	3 (13.6)	5 (22.7)	5 (22.7)	2 (9.1)	4 (18.4)	0.2
Vocational	103 (9.3)	9 (8.7)	41 (39.8)	16 (15.5)	8 (7.8)	20 (19.4)	9 (8.7)	
Secondary	486 (43.7)	59 (12.1)	173 (35.6)	97 (20.0)	51 (10.5)	73 (15.0)	33 (6.8)	
Higher	502 (45.1)	60 (12.0)	167 (33.3)	112 (22.3)	53 (10.6)	85 (16.9)	25 (5.0)	
Married								
Yes	593 (53.3)	84 (14.2)	223 (37.6)	121 (20.4)	66 (11.1)	84 (14.2)	15 (2.5)	<0.001
No	520 (46.7)	47 (9.0)	161 (31.0)	109 (21.0)	51 (9.8)	96 (18.5)	56 (10.8)	
Have children								
Yes	760 (68.3)	101 (13.3)	294 (38.7)	162 (21.3)	78 (10.3)	108 (14.2)	17 (2.2)	<0.001
No	353 (31.7)	30 (8.5)	90 (25.5)	68 (19.3)	39 (11.0)	72 (20.4)	54 (15.3)	
Number of household members								
Living alone	189 (17.0)	16 (8.5)	62 (32.8)	41 (21.7)	20 (10.6)	29 (15.3)	21 (11.1)	0.06
2 or more	924 (83.0)	115 (12.4)	322 (34.8)	189 (20.5)	97 (10.5)	151 (16.3)	50 (5.4)	
Location of place of residence								
Rural area	438 (39.4)	53 (12.1)	133 (30.4)	78 (17.8)	53 (12.1)	84 (19.2)	37 (8.4)	0.07
City with <20,000 residents	131 (11.8)	10 (7.6)	52 (39.7)	23 (17.6)	14 (10.7)	21 (16.0)	11 (8.4)	
City with ≥20,000 and <99,999 residents	224 (20.1)	28 (12.5)	85 (37.9)	56 (25.0)	20 (8.9)	25 (11.2)	10 (4.5)	
City with ≥100,000 and <499,999 residents	183 (16.4)	23 (12.6)	62 (33.9)	44 (24.0)	21 (11.5)	27 (14.8)	6 (3.3)	
City with ≥500,000 residents	137 (12.3)	17 (12.4)	52 (38.0)	29 (21.2)	9 (6.6)	23 (16.8)	7 (5.1)	
Occupational status								
Active	629 (56.5)	65 (10.3)	200 (31.8)	141 (22.4)	67 (10.7)	108 (17.2)	48 (7.6)	0.03
Passive	484 (43.5)	66 (13.6)	184 (38.0)	89 (18.4)	50 (10.3)	72 (14.9)	23 (4.8)	
Financial status								
Good	523 (47.0)	54 (10.3)	187 (35.8)	101 (19.3)	57 (10.9)	87 (16.6)	37 (7.1)	0.8
Moderate	418 (37.6)	56 (13.4)	141 (33.7)	93 (22.2)	44 (10.5)	62 (14.8)	22 (5.3)	
Bad	172 (15.5)	21 (12.2)	56 (32.6)	36 (20.9)	16 (9.3)	31 (18.0)	12 (7.0)	
Presence of chronic diseases								
Yes	468 (42.0)	81 (17.3)	202 (43.2)	83 (17.7)	40 (8.5)	53 (11.3)	9 (1.9)	<0.001
No	645 (58.0)	50 (7.8)	182 (28.2)	147 (22.8)	77 (11.9)	127 (19.7)	62 (9.6)	
Visit to a doctor due to urologic diseases in the last 6 months								
Yes	162 (14.6)	45 (27.8)	74 (45.7)	21 (13.0)	10 (6.2)	6 (3.7)	6 (3.7)	<0.001
No	951 (85.4)	86 (9.0)	310 (32.6)	209 (22.0)	107 (11.3)	174 (18.3)	65 (6.8)	

**Table 2:** Factors associated with the frequency of urinalysis in a representative sample of adults in Poland, March 2023 (*n* = 1113).

Variables	Urinalysis in the Last 12 Months				Urinalysis in the Last 3 Years			
			Univariable Logistic Regression	Multivariable Logistic Regression			Univariable Logistic Regression	Multivariable Logistic Regression
	*n* (%)	*p*	OR (95%CI)	OR (95%CI)	*n* (%)	*p*	OR (95%CI)	OR (95%CI)
Gender								
Female	287 (49.1)	0.04	1.28 (1.01–1.62) *	1.31 (1.01–1.68) *	482 (82.5)	<0.001	1.85 (1.39–2.47) ***	1.92 (1.42–2.62) ***
Male	228 (43.1)		Reference	Reference	380 (71.8)		Reference	Reference
Age [years]								
18–29	61 (35.3)	<0.001	Reference	Reference	109 (63.0)	<0.001	Reference	Reference
30–39	69 (32.1)		0.87 (0.57–1.33)	0.63 (0.39–1.00)	146 (67.9)		1.24 (0.82–1.89)	0.83 (0.52–1.34)
40–49	74 (40.0)		1.22 (0.80–1.88)	0.79 (0.49–1.29)	143 (77.3)		2.00 (1.26–3.17) **	1.20 (0.71–2.04)
50–59	72 (51.8)		1.97 (1.25–3.11) **	1.04 (0.61–2.16)	110 (79.1)		2.23 (1.33–3.72) **	1.07 (0.58–1.96)
60–69	148 (55.6)		2.30 (1.55–3.42) ***	1.31 (0.79–2.16)	227 (85.3)		3.42 (2.16–5.41) ***	1.85 (1.03–3.32) *
70 and over	91 (67.4)		3.80 (2.36–6.11) ***	2.22 (1.23–4.02) **	127 (94.1)		9.32 (4.28–20.30) ***	5.67 (2.35–13.64) ***
Education								
Primary	6 (27.3)	0.3	Reference		16 (72.7)	0.5	Reference	
Vocational	50 (48.5)		2.52 (0.91–6.94)		74 (71.8)		0.96 (0.34–2.69)	
Secondary	232 (47.7)		2.44 (0.94–6.33)		380 (78.2)		1.34 (0.51–3.52)	
Higher	227 (45.2)		2.20 (0.85–5.72)		392 (78.1)		1.34 (0.51–3.50)	
Married								
Yes	307 (51.8)	<0.001	1.61 (1.27–2.04) ***	1.34 (0.99–1.80)	494 (83.3)	<0.001	2.06 (1.55–2.75) ***	1.43 (0.98–2.08)
No	208 (40.0)		Reference	Reference	368 (70.8)		Reference	Reference
Have children								
Yes	395 (52.0)	<0.001	2.10 (1.62–2.73) ***	1.45 (1.01–2.09) *	636 (83.6)	<0.001	2.82 (2.11–3.77) ***	1.65 (1.08–2.08) *
No	120 (34.0)		Reference	Reference	227 (64.3)		Reference	Reference
Number of household members								
Living alone	78 (41.3)	0.1	Reference		139 (73.5)	0.2	Reference	
2 or more	437 (47.3)		1.28 (0.93–1.75)		723 (78.2)		1.29 (0.90–1.85)	
Location of place of residence								
Rural area	186 (42.5)	0.3	Reference		317 (72.4)	0.01	Reference	Reference
City with <20,000 residents	62 (47.3)		1.22 (0.82–1.80)		99 (75.6)		1.18 (0.75–1.85)	1.04 (0.64–1.69)
City with ≥20,000 and <99,999 residents	113 (50.4)		1.38 (0.99–1.91)		189 (84.4)		2.06 (1.36–3.13) ***	1.84 (1.19–2.86)
City with ≥100,000 and <499,999 residents	85 (46.4)		1.18 (0.83–1.66)		150 (82.0)		1.74 (1.13–2.67) **	1.48 (0.93–2.34)
City with ≥500,000 residents	69 (50.4)		1.38 (0.94–2.02)		107 (78.1)		1.36 (0.86–2.15)	1.21 (0.75–1.98)
Occupational status								
Active	265 (42.1)	0.002	Reference	Reference	473 (75.2)	0.04	Reference	1.57 (1.08–2.27) *
Passive	250 (51.7)		1.47 (1.16–1.86) **	0.76 (0.56–1.05)	389 (80.4)		1.35 (1.01–1.80) *	Reference
Financial status								
Good	241 (46.1)	0.9	1.05 (0.75–1.49)		399 (76.3)	0.3	1.07 (0.72–1.60)	
Moderate	197 (47.1)		1.10 (0.77–1.57)		334 (79.9)		1.33 (0.87–2.02)	
Bad	77 (44.8)		Reference		129 (75.0)		Reference	
Visit to a doctor due to urologic diseases in the last 6 months								
Yes	283 (60.5)	<0.001	2.72 (2.13–3.48) ***	2.34 (1.79–3.02) ***	406 (86.8)	<0.001	2.71 (1.98–3.73) ***	2.13 (1.51–3.01) ***
No	232 (36.0)		Reference	Reference	456 (70.7)		Reference	Reference

* *p* < 0.05; ** *p* < 0.01; *** *p* < 0.001.

**Table 3:** Factors associated with the frequency of urinalysis among adults without urologic diseases in Poland, March 2023 (*n* = 951).

Variables	Urinalysis in the Last 12 Months				Urinalysis in the Last 3 Years			
			Univariable Logistic Regression	Multivariable Logistic Regression			Univariable Logistic Regression	Multivariable Logistic Regression
	*n* (%)	*p*	OR (95%CI)	OR (95%CI)	*n* (%)	*p*	OR (95%CI)	OR (95%CI)
Gender								
Female	228 (44.8)	0.03	1.32 (1.02–1.72) *	1.33 (1.02–1.74) *	412 (80.9)	<0.001	2.01 (1.49–2.71) ***	2.03 (1.48–2.79) ***
Male	168 (38.0)		Reference	Reference	300 (67.9)		Reference	Reference
Age [years]								
18–29	49 (32.2)	<0.001	Reference	Reference	93 (61.2)	<0.001	Reference	Reference
30–39	51 (27.9)		0.81 (0.51–1.30)	0.69 (0.42–1.12)	118 (64.5)		1.15 (0.74–1.80)	0.83 (0.51–1.36)
40–49	60 (36.6)		1.21 (0.76–1.93)	0.99 (0.60–1.61)	123 (75.0)		1.90 (1.18–3.08) **	1.28 (0.74–2.21)
50–59	55 (46.6)		1.84 (1.12–3.02) *	1.45 (0.85–2.45)	90 (76.3)		2.04 (1.19–3.48) **	1.21 (0.65–2.27)
60–69	120 (51.5)		2.23 (1.46–3.42) ***	2.03 (1.26–3.26) **	194 (83.3)		3.16 (1.96–5.07) ***	2.01 (1.10–3.68) *
70 and over	61 (60.4)		3.21 (1.90–5.41) ***	3.29 (1.84–5.87) ***	94 (93.1)		8.52 (3.70–19.62) ***	6.68 (2.64–16.88) ***
Education								
Primary	5 (26.3)	0.6	Reference		15 (78.9)	0.4	Reference	
Vocational	35 (42.7)		2.09 (0.69–6.33)		55 (67.1)		0.54 (0.16–1.80)	
Secondary	176 (42.2)		2.05 (0.72–5.78)		315 (75.5)		0.82 (0.27–2.54)	
Higher	180 (41.6)		1.99 (0.71–5.63)		327 (75.5)		0.82 (0.27–2.53)	
Married								
Yes	236 (46.9)	<0.001	2.18 (1.63–2.92) ***	1.45 (1.09–1.94) *	407 (80.9)	<0.001	1.99 (1.48–2.68) ***	1.42 (0.96–2.10)
No	160 (35.7)		Reference	Reference	305 (68.1)		Reference	Reference
Have children								
Yes	308 (47.4)	<0.001	0.76 (0.54–1.08)		528 (81.2)	<0.001	2.75 (2.03–3.73) ***	1.55 (0.99–2.43)
No	88 (29.2)		Reference		184 (61.1)		Reference	Reference
Number of household members								
Living alone	59 (36.2)	0.1	0.76 (0.54–1.08)		115 (70.6)	0.2	0.7 (0.53–1.11)	
2 or more	337 (42.8)		Reference		597 (75.8)		Reference	
Location of place of residence								
Rural area	145 (38.3)	0.2	0.76 (0.50–1.15)		265 (69.9)	0.02	Reference	Reference
City with <20,000 residents	52 (44.1)		0.97 (0.58–1.62)		88 (74.6)		1.26 (0.79–2.02)	1.25 (0.76–2.06)
City with ≥20,000 and <99,999 residents	86 (47.5)		1.11 (0.70–1.77)		149 (82.3)		2.00 (1.29–3.11) *	1.58 (0.99–2.53)
City with ≥100,000 and <499,999 residents	60 (38.7)		0.78 (0.48–1.26)		122 (78.7)		1.59 (1.02–2.48) *	1.91 (1.20–3.03) **
City with ≥500,000 residents	53 (44.9)		Reference		88 (74.6)		1.26 (0.79–2.02)	1.17 (0.71–1.93)
Occupational status								
Active	208 (38.3)	0.02	Reference	Reference	392 (72.2)	0.03	Reference	Reference
Passive	188 (46.1)		1.38 (1.06–1.79) *	0.78 (0.59–1.10)	320 (78.4)		1.40 (1.04–1.89) *	0.73 (0.50–1.07)
Financial status								
Good	199 (43.2)	0.7	1.13 (0.77–1.66)		343 (74.4)	0.4	1.18 (0.78–1.79)	
Moderate	140 (40.2)		1.00 (0.67–1.50)		268 (77.0)		1.36 (0.88–2.11)	
Bad	57 (40.1)		Reference		101 (71.1)		Reference	

* *p* < 0.05; ** *p* < 0.01; *** *p* < 0.001.

## Data Availability

Data are available from the corresponding author on reasonable request.

## Supplementary Materials

The following supporting information can be downloaded at: https://www.mdpi.com/article/10.3390/healthcare12151475/s1, File S1: Study questionnaire.
