# Supplementary material for: A Cross-Sectional Survey to Identify Sociodemographic Factors Associated with the Frequency of Urinalysis in a Representative Sample of Adults in Poland, 2024
Source: Healthcare (Basel). 2024 Jul 25;12(15):1475. doi: 10.3390/healthcare12151475 (PMC11311361; doi:10.3390/healthcare12151475)
Supplement: Supplementary file 1 [file healthcare-12-01475-s001.zip › healthcare-3077245-supplementary.pdf]

STUDY QUESTIONNAIRE

Read the description below and then answer the questions.

Urinary tract diseases are one of the most common health ailments. Knowledge about urinary tract diseases allows you to significantly reduce the risk of their occurrence and respond quickly in the event of disturbing symptoms.

The rest of the survey contains questions that will help develop the assumptions for the prevention and education program about urinary tract diseases in Poland.

[P1] What do you think are the risk factors for kidney cancer? Select all that you think may lead to kidney cancer.

- Smoking tobacco, cigarettes
- Obesity
- Male gender
- Older age
- Low level of physical activity (e.g. sedentary lifestyle)
- Hypertension
- Exposure to certain chemical agents (heavy metals, tannins, asbestos)
- Genetic predispositions, e.g. occurrence of kidney cancer in related family members
- Chronic use of painkillers
- Long-term treatment of kidney disease with dialysis
- None of these
- It's hard to say

[P2] What do you think are the risk factors fobladder cancer? Select all that you think may lead to bladder cancer.

- Exposure to chemical substances, e.g. when working in the production of paints, dyes, aluminum
- Arsenic contamination of drinking water
- Obesity
- Older age
- Male gender
- A diet e.g. high in meat
- Smoking tobacco, cigarettes
- Recurrent bladder infections
- Genetic predispositions, e.g. occurrence of bladder cancer in related family members
- Previous radiotherapy or chemotherapy (with cyclophosphamide or ifosfamide)
- None of these
- It's hard to say

[Q3] What do you think are the risk factors for prostate cancer? Select all that you think may lead to bladder cancer.

- Older age
- Genetic predisposition, e.g. occurrence of prostate cancer in related family members
- Smoking tobacco, cigarettes
- Obesity
- Metabolic syndrome
- Diet (high consumption of meat and dairy products)
- Decreased sexual activity
- Exposure to chemicals such as pesticides and cadmium (e.g. farmers, welders, heavy industry workers)
- None of these
- It's hard to say

[Q4] Which of the following symptoms do you think may be symptoms of bladder cancer? Check all that you think may be symptoms of bladder cancer.

- Hematuria (presence of blood in urine)
- Frequent, painful or difficult urination
- A feeling of constant pressure on the bladder or incomplete emptying of the bladder
- Pain in the lower abdomen, suprapubic or lumbar region
- A palpable lump or enlarged lymph nodes in the bladder area
- Bone pain
- Swelling of the lower limbs (legs)
- None of these
- It's hard to say

[P5] In your opinion, which of the following behaviors may lead to the development of urolithiasis, i.e. the formation of deposits (so-called "stones") in the kidneys or urinary tract? Select all those that you think may lead to the development of kidney stones.

- Obesity
- Lipid disorders (e.g. high cholesterol)
- Type 2 diabetes
- Drinking highly mineralized water
- Drinking too little water
- A diet high in protein or high in oxalates (e.g. spinach, sorrel, Swiss chard, soybeans, rhubarb)
- Genetic predispositions, e.g. occurrence of kidney stones in related family members
- Use of selected groups of drugs (e.g. corticosteroids and alkalizing preparations)
- None of these
- It's hard to say

[Q6] Do you have chronic diseases or long-term health problems lasting at least 6 months?

- Yes
- No

[P7] In the last 6 months, have you had any urologic diseases (e.g., kidneys, bladder, pros-tate diseases, urinary tract infections) for which you consulted a doctor?

- Yes
- No

[Q8] When was the last time you had a urine test (passed urine for analysis)?

- Over the last month
- Over a month ago but no more than 12 months ago
- Over a year ago but no more than 2 years ago
- More than 2 years ago but not more than 3 years ago
- Over 3 years ago
- Never

Socio-metric questions

1. What is you gender?

- woman
- man

2. How old are you?

________________________ [age in years]

3. What is the size of the town you live in?

- village
- small town (up to 20,000 inhabitants)
- medium-sized city (20,000 to 99,000 inhabitants)
- large city (between 100,000 and 500,000 inhabitants)
- large city (more than 500,000 inhabitants)

__________ please insert your postal code

4. What is your current education (most recently completed school)?

- primary or lower secondary school
- essential
- medium
- post-secondary or university degree
- bachelor degree
- completed university degree

5. How would you rate your household financial situation?

- good
- moderate
- bad

7. What is your current professional status?

- I work under an employment contract
- I work on a civil contract basis
- I am self-employed
- unemployed
- pensioner
- pupil or student
- I am in charge of housekeeping
- other (provide details)

8. What is your marital status?

- single
- married
- in an informal relationship
- other (provide details)

9. Do you have children under 18 living with you?

- yes
- not

10. In which province do you live?

- Dolnośląskie
- Kujawsko-Pomorskie
- Lublin
- Lubuskie
- Lodz
- Małopolskie
- Mazowsze
- Opolskie
- Podkarpackie
- Podlaskie
- Pomeranian
- Śląskie
- Świętokrzyskie
- Warminsko-Mazurskie
- Wielkopolskie
- Zachodniopomorskie
